# The latent profile analysis of Chinese adolescents’ gaming disorder: examination and validation

**DOI:** 10.1186/s12888-023-05320-8

**Published:** 2023-11-13

**Authors:** Lina Zhang, Mengqi Liu, Ming Yuan, Mutian Hou, Cheng Yang, Yingying Wang, Wei Hao, Yanhui Liao

**Affiliations:** 1 Department of Clinical Psychology, Tianjin Medical University General Hospital, Tianjin, China; 2 The National Clinical Research Center for Mental Disorders and Beijing Key Laboratory of Mental Disorders and Beijing Institute for Brain Disorders Center of Schizophrenia, Beijing Anding Hospital, Capital Medical University, Beijing, China; 3 Advanced Innovation Center for Human Brain Protection, Capital Medical University, Beijing, China; 4 Department of Applied Psychology, Hunan University of Chinese Medicine, Changsha, China; 5 Psychological Research and Counseling Center, Southwest Jiaotong University, Chengdu, China; 6 Department of Psychiatry, National Clinical Research Center for Mental Disorders, the Second Xiangya Hospital of Central South University, Changsha, China; 7 School of Physical Education and Health, Hunan University of Technology and Business, Changsha, China; 8 Department of Psychiatry, Sir Run Run Shaw Hospital, School of Medicine, Zhejiang University, Hangzhou, China

**Keywords:** Latent profile analysis, Chinese adolescents, Gaming disorder

## Abstract

**Background:**

Gaming disorder is a new disease, which is included in the disease unit of disorder caused by addiction in the 11th revision of the International Classification of Diseases. This study examined the symptom characteristics of gaming disorders in Chinese adolescents using the latent profile analysis.

**Methods:**

Totally, 5988 students (including 3285 boys and 2703 girls; aged 12–18 years) from junior high schools and senior high schools were enrolled. The Gaming Disorder Symptom Questionnaire-21 (GDSQ-21) was used to screen gaming disorder. A latent profile analysis was used for classifying the subgroups based on the extent of gaming usage. The relationship between adolescent gamers and demographic variables was analyzed by logistic regression.

**Results:**

The results of latent profile analysis supported the models of four latent profiles, which were defined as healthy gamers (Profile 1, 56.83%), impaired control gamers (Profile 2, 26.09%), impaired control-game priority gamers (Profile 3, 9.72%) and gamers with disorder (Profile 4, 7.36%), respectively. Logistic regression analysis found that, compared with girls, boys were more likely to be classified into the group dominated by the impaired gamers, the impaired control-game priority gamers, and the gamers with disorder.

**Conclusions:**

This study highlighted that the latent profile analysis identified four different groups of adolescent gamers, showing a clearer conceptualization of heterogeneous gamers. Gender and average weekly gaming time can predict the latent profile of adolescents. Our findings may facilitate the design of individualized assessment and early intervention programs for adolescent gamer users based on different gaming usage symptoms.

**Supplementary Information:**

The online version contains supplementary material available at 10.1186/s12888-023-05320-8.

## Background

### Definition of gaming disorder (GD)

Following a provisional status for Internet gaming disorder (IGD) in Section III of the Diagnostic and Statistical Manual of Mental Disorders, 5th Edition (DSM-5) [1], GD was officially adopted at the World Health Assembly in May 2019 as a diagnosis in the International Classification of Diseases, 11th Revision (ICD-11). GD within the ICD-11 is characterized by impaired control over gaming, persistent gaming behavior, and functional impairment, the criteria concerning a recurrent on-/off-line usage pattern (“digital gaming” or “video gaming”) that is usually present over a period of at least 12 months in most instances [2]. In fact, moderate gaming behaviors can be a pleasant and relaxing experiences for most adolescents, such as socializing, relieving stress and loneliness [3]. However, a minority of adolescents can be unable to regulate or cease their excessive gaming behavior, resulting in disruption of normal daily life and basic self-care (i.e., meals, sleep time, personal hygiene) [3, 4]; relationships and important responsibilities (i.e., homework, or academics) [5, 6]. However, some individuals have pathological cognitive-behavioral gaming symptoms but no severe functional impairment. Therefore, it is not appropriate to over-pathologize all gamers as a homogeneous monolith of GD [7], as the main goal of the present study has focused on distinguishing the different characteristics of individuals of gamers.

### Etiology of GD

The etiology of GD is complex. Now, there are several important factors associated with gaming disorder: Neurobiology anomalies (i.e., altered grey matter volume, functional connectivity, and activation in specific brain regions) [8]; psychopathological anomalies (i.e., impulsivity, poor self-efficacy and low self-esteem) [9]; Familial factors(i.e., parenting styles, parent-child relationship, and family cohesion) [10]; and social factors (i.e., absence of social support and insufficient interpersonal relationships) [11]; In addition, there is also a strong link between Game-related factors game motivation and negative game outcomes [12].

### Symptom heterogeneity of GD

Most of the previous studies [13, 14] have investigated GD in adolescents using the dimensions and scores in the questionnaires of GD, that is, statistical analysis is conducted based on different levels or classifications of variables. This is helpful for us to understand the relationship between key variables and GD. However, at the individual level, each patient has his/her own characteristics in multiple variables, and these variables play a combined role in the individual gaming behavior. If we only understand the role of variables, it will be difficult to describe the heterogeneity between individuals or subgroups, which is relatively fragmented for the understanding of individuals with online or offline gaming usage.

The research taking individuals as the center is conducive to more clearly portraying the details of heterogeneous gamers, and is helps conduct targeted research on different subgroups of online or offline gamers. Latent profile analysis (LPA) is one of the statistical methods to achieve person-centered researches [15]. Therefore, the classification of individual latent characteristics is evaluated according to the response mode of the individual on the explicit test items, and the accuracy and effectiveness of classification are measured by objective statistical indicators [16]. To ensure the maximization of inter-group heterogeneity and intra-group homogeneity, LPA is complementary to other latent structural analysis methods such as factor analysis, which can more comprehensively reveal the inherent nature of heterogeneous gamers.

### Correlates of GD

More time spent on the gaming is a significant predictor of GD is also an important discussion [17]. The results of more studies showed that GD is related to daily gaming time [18–20]. Studies have also investigated the association of age and gender with IGD. In terms of age, although the effect of age on the prevalence of GD is not clear from most studies, only a minority of studies so far has reported finding that the highest prevalence of IGD was found in adolescents [21]. Findings are more consistent with respect to gender, with male adolescents having a higher prevalence of GD [22].

## Aims of the current study

The current study intended to identify homogenous subtypes of heterogeneous gamers among Chinese adolescents, that is, the different characteristics of individuals with online or stand-alone gamers. Firstly, LPA was used to analyze the latent structure of gamers and the latent characteristics of each profile. Secondly, the present study analyzed age and gender differences among different latent profiles of heterogeneous online or stand-alone gamers. We hypothesize that distinct latent profiles of heterogeneous gamer symptoms may emerge based on severity levels, although we expect those different severity subgroups to demonstrate different patterns of heterogeneous gamer themes. Additionally, compared with girls, more boys would be classified in the group with highly engaged gaming.

## Methods

### Participants

This cross-sectional survey was conducted in three Chinese cities (i.e., Urumqi, Kashi and Bole in Xinjiang Uygur Autonomous Region, China), with a total of 7,901 participants. After excluding cases due to missing values, the valid sample consisted of 7790 students, with an effective rate of 98.6%. Among them, 5988 internet or video gamers whose mean age ranged from 12 to 18 years (mean = 14.98 years, *SD* = 1.63 years), and 54.9% were males (n = 3285). Table 1 shows the sociodemographic information.

**Table 1:** Sociodemographic characteristics of the study participants

Sociodemographic characteristics	Total
Age, years; mean (SD)	14.98 (1.63)
Gender (males, n, %)	3285 (54.9)
School (n, %)	
Junior high school	2726 (45.5)
Senior high school	3262 (54.5)
Family structure (n, %)	
Being an only child	2934 (49.0)
Having siblings	3054 (51.0)
The highest education level of the family (n, %)	
Primary school or below	199 (3.3)
Junior high school	1254 (20.9)
Senior high school or vocational high school	1429 (23.9)
College or junior college	2788 (46.6)
Master or above	318 (5.3)
Family occupational stratum ^a^ (n, %)	
1 ~ 2	377 (6.3)
3 ~ 4	1181 (19.7)
5 ~ 6	3045 (50.9)
7 ~ 8	1208 (20.2)
9 ~ 10	177 (3.0)
Types of games most often played (n, %)	
Massively Multiplayer Online Games (MOBA)	2097 (35.0)
First-person shooter games (FPS)	703 (11.7)
Building and management games (My World)	434 (7.2)
Massively multiplayer online role-playing games (MMORPG)	324 (5.4)
Sports Games (FIFA)	298 (5.0)
Etc	2132 (35.7)
Weekly gameplay (n, %)	
Less than 2 h	3386 (56.5)
Between 2 and 4 h	1044 (17.4)
Between 4 and 8 h	756 (12.6)
Between 8 and 16 h	366 (6.1)
Between 16 and 32 h	202 (3.4)
Between 32 and 64 h	110 (1.8)
Between 64 and 128 h	69 (1.2)
More than 128 h per week	55 (0.9)

^*a*^*Family occupational stratum* 1 means the lowest and 10 means the highest

### Measures

#### Sociodemographic data collection

The sociodemographic data were collected with a self-designed questionnaire. The demographic data included age, gender, grade, and family structure (having siblings or being an only child). The sociological information related to family included the highest level of education of family members and family occupational stratum.

#### Gameplay pattern

The survey asked questions concerning the types of games most often played, the weekly time spent playing on primarily online, stand-alone and/or video games and distinguished between those that played less than 2 h, between 2 and 4 h, between 4 and 8 h, between 8 and 16 h, between 16 and 32 h, between 32 and 64 h, between 64 and 128 h, and more than 128 h per week respectively.

#### Gaming disorder symptom questionnaire-21 (GDSQ-21) [23]

GDSQ-21 is one of the measurement tools for screening GD in ICD-11. The 5-Likert scale was used for scoring (0 = never, 1 = less than monthly, 2 = monthly, 3 = weekly, and 4 = almost daily). There were 21 items and 3 dimensions, including impaired control gaming behaviors (impaired control), increasing priority to gaming over other life interests and daily activities (increasing priority), and continuous gaming regardless of obvious functional impairment and negative consequences (continuous). It has good validity and internal consistency reliability, with the Cronbach’s α coefficient of 0.964, and plays an important role in the investigation of GD.

### Procedure

Researchers first contacted potential schools to collaborate on data collection, and then twelve schools agreed to participate in the current study. The period of the data collection spanned from October 2020 to November 2021, and the data in the current survey are only a part of a big set of studies that contained multiple questionnaires. Firstly, the study will respect and protect the subjects’ right to decide whether to participate in the study and strictly fulfill the informed consent procedure; secondly, all the contents of the survey will be clearly explained to the students and their parents before the survey, and the parents will fill in the informed consent form after obtaining the consent of the students and their parents. After going through the consent process, the adolescents were asked to complete a set of questionnaires including a demographic information survey, a survey of game use patterns and risky behaviors, the GDSQ-21. The survey was carried out in the classrooms of the recruited classes. Students were then allowed 30 min to complete the self-report questionnaires. Two research assistant was present to provide clarification and explained the study purpose and the items in the questionnaires.

### Statistical analyses

Mplus 8.3 software was used for statistical analysis. The maximum likelihood estimation with robust standard error was used for LPA and the most likely number of profiles based on GDSQ-21 dimensions was explored. LPA uses latent category variables to explain the relationship between explicit variables (dependent variables). The model fitness test was performed. The optimal model was selected. The conditional probability and profile probability based on the optimal model were calculated.

To determine the optimal number of latent profiles in the test and verification samples, each model was evaluated using the following fitting indicators: Akaike information criteria (AIC) [24], adjusted Bayesian Information Criterion (aBIC) [25], Bayesian Information Criterion (BIC) [26], Parametric Bootstrap Likelihood Ratio Test (BLRT) [26], and Lo Mandell Rubin Likelihood Ratio Test (LMR-LRT). According to the previous description by Nylund et al. [27], BLRT was the optimal evaluation indicator, followed by BIC, aBIC and Entropy values. The smaller the AIC, BIC, and aBIC values were, the better the model fit was. BLRT and LMR both could generate *p* values to evaluate whether the models of *k* profiles fit better than those of *k-1* profiles, in which *k* = number of profiles. The higher classification discrimination indicated a smaller classification error [27]. The precision of latent profile membership assignment was generally represented by the index Entropy. The higher Entropy value indicated a more accurate classification. The Entropy value greater than 0.80 indicated that the latent profile was highly recognized, and when the Entropy value was close to 1.0, a clearer result will be displayed. In addition to using the fitting index to determine the optimal profile of the test and verification samples, the study also tested the competition model based on the group classification probability (posterior classification probability) of the most likely profile. The posterior classification probability ranged from 0 to 1 and had a high diagonal value, indicating that the model has a higher confidence level.

To test the predictive effect of gender and age on different latent profiles of GD symptoms, we constructed the mixture regression model [28] based on the demographic variables and gaming time for online, stand-alone, and video games. Multiple Logistic regression was conducted with LPA results as dependent variables, gender and age as independent variables, and the OR (odds ratio) was analyzed.

## Results

### LPA model of GD

The LPA was established by taking the scores of the three dimensions of GDSQ-21 as the explicit variables, and the latent profiles were set to 5-profile model. Table 2 shows the fitting indexes of different profiles (from the 1-profile model to the 5-profile model). After comparison of the 1-to-5 profile model, we found that the LMR-LRT value was not significant in the 5-profile model (*p* = .535 > .05), indicating that the 5-profile model was not better than the 4-profile model. For the 4-profile models, LMR-LRT and BLRT confirmed that LPA had good adaptability (*p* < .001). Compared with the 3-profile model, the 4-profile model showed lower AIC, BIC and aBIC values, and Entropy was 0.945. Therefore, we selected the 4-profile model as the optimal model.

**Table 2:** Fitting index and class probability of five category models for latent profile analysis of GDSQ-21

Model	K	Log (Likelihood)	AIC	BIC	a BIC	Entropy	Significance test		Class probability (%)				
							LMR LRT *p*-value	BLRT *p*-value	1	2	3	4	5
1	42	-184514.971	369113.941	369395.237	369261.773	--	--	--	1				
2	64	-154229.540	308587.081	**309015.722**	308812.348	**0.991**	< 0.001	< 0.001	**0.854**	**0.146**			
3	86	-141799.937	283771.874	284347.860	284074.577	0.979	< 0.001	< 0.001	0.751	0.174	0.074		
4	108	-136875.519	273967.037	274690.369	**274347.175**	0.945	< 0.001	< 0.001	0.570	0.259	0.097	0.073	
5	130	-133273.665	**266807.331**	267678.007	267264.904	0.952	0.535	< 0.001	0.0456	0.259	0.559	0.083	0.052

* K* number of freely estimated parameters, *AIC* Akaike information criteria, *aBIC* adjusted Bayesian Information Criterion, *Entropy* Classification accuracy index, *BLRT* Bootstrap Likelihood Ratio Test, *LMR-LRT* Lo Mandell Rubin Likelihood Ratio Test

Table 3 shows the attribution probability matrix of the four-profile pattern (97.6%, 93.3%, 98.0% and 99.2%). The average probabilities of Profile 1, Profile 2, Profile 3 and Profile 4 in the 4-profile model were, respectively, which indicates that the LPA of the 4-profile model has good identifiability and reliability.

**Table 3:** Average attribution probability (average posterior probability) of the most likely category members (rows) by latent category (column)

Category	Attribution probability			
	Profile 1 (n = 3403)	Profile 2 (n = 1562)	Profile 3 (n = 582)	Profile 4 (n = 441)
Profile 1	0.976	0.024	0.000	0.000
Profile 2	0.060	0.933	0.006	0.000
Profile 3	0.000	0.017	0.980	0.003
Profile 4	0.000	0.002	0.006	0.992

The columns indicate the latent category and the rows indicate the most likely category members

**Fig. 1:**
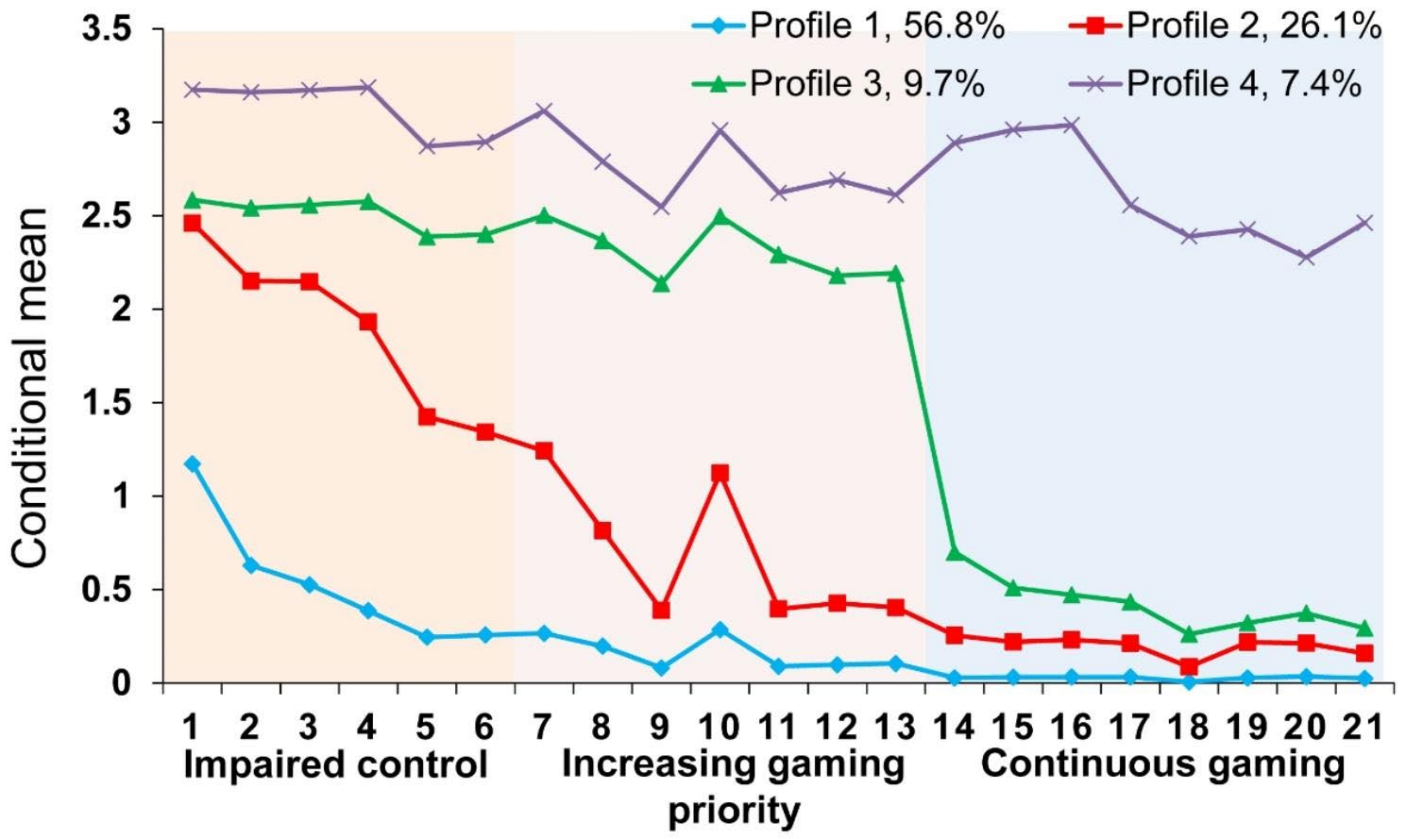
Distribution of the estimated conditional mean of 4 latent classes

Figure 1 visualizes the estimated conditional mean of the 4-profile model on 21 items. The results showed that the scoring probabilities of the 4-latent profile model on 21 items of GD symptoms differed significantly and showed different characteristics. Then we divided the populations with gaming usage into 4 profiles. Supplementary Table 1 shows the mean values of each profile in each item.

Table 4 shows the mean values comparing of different profiles of adolescents in various dimensions of GDSQ-21. Adolescents in Profile 1 had the lowest scores in the dimensions of impaired control (*M* = 3.19), increasing priority (*M* = 1.07), and continuation (*M* = 0.21). There were significant differences between impaired control gaming behavior and the other two dimensions (*ps* < 0.001). According to its scoring characteristics, adolescents in Profile 1 were named healthy gamers (56.8%). The Profile 2 scored the highest in the dimension of impaired control (*M* = 11.42), followed by increasing priority (*M* = 4.88) and continuation (*M* = 1.58). There was a significant difference between impaired control gaming behavior and other dimensions (*ps* < 0.001). The adolescents in Profile 2 accounted for 26.1% of all adolescents and were named impaired control gamers (26.1%). The average score of Profile 3 in the dimensions of impaired, increasing priority and continuation was higher than that of Profile 2 and was named impaired control - game priority gamers (9.7%). The conditional probability of Profile 4 in 21 items was significantly higher than that of the other three profiles, indicating that the adolescents in Profile 4 had the highest level of GD compared with the other three profiles and was named gamers with disorder (7.4%). The average score of impaired control, increasing priority and, continuation was 18.45, 19.27, and 20.93, respectively. There were statistically significant differences among the four profiles of adolescents in the total score of GD symptoms and the scores of each dimension (*P* < .001). With the increase in the risk of GD, the total score of GDSQ-21 and the scores of each dimension in adolescents were also significantly increased.

**Table 4:** Comparison of mean values of different categories of adolescents in various dimensions of GDSQ-21

Variable	Profile 1	Profile 2	Profile 3	Profile 4	F	${\rm{\eta}}_{\rm{p}}^{\rm{2}}$	Group differences
	n = 3403(56.83%)	n = 1562(26.09%)	n = 582(9.72%)	n = 441(7.36%)	-	-	-
Impaired control	3.19 ± 2.47	11.42 ± 3.70	15.05 ± 4.11	18.45 ± 3.95	5863.507^***^	0.746	P1 < P2 < P3 < P4
Increasing priority	1.07 ± 1.73	4.88 ± 3.11	16.16 ± 4.01	19.27 ± 5.82	8653.224^***^	0.813	P1 < P2 < P3 < P4
Continuous	0.21 ± 0.98	1.58 ± 2.87	3.35 ± 3.85	20.93 ± 5.36	9007.412^***^	0.819	P1 < P2 < P3 < P4
GDSQ-21	4.47 ± 3.50	17.88 ± 5.47	34.56 ± 7.46	58.65 ± 11.7	16008.944^***^	0.889	P1 < P2 < P3 < P4

*Profile 1* healthy gamers, *Profile 2* impaired control gamers, *Profile 3* impaired control game priority gamers, *Profile 4* gamers with disorder, ***p < .001, **p < .01, *p < .05

### The predictive effect of demographic variables on 4 latent profiles

Table 5 showed the results of multiple Logistic regression that gender, age, family structure and socio-economic status of the family could help predict the latent profile of adolescents GD. Compared with Profile 1, the OR of girls to become Profile 2, Profile 3 and Profile 4 was 96.8%, 71.4%, and 62.2% lower. The age of adolescents could also predict the latent profile of adolescents. With Profile 1 as the reference group, with the increase of age, the OR of adolescents to become Profile 2 was 18.6% higher. Compared to being an only child, the OR of having siblings to become Profile 2 was 23.0% lower. With the increase of education level of the family and occupational stratum, the OR of these gamers to become Profile 3 was 18.5% and 15.2% lower.

**Table 5:** Multiple Logistic regression results of demographic variables in 4 latent profiles

Variable	Profile 2 VS Profile 1		Profile 3 VS Profile 1		Profile 4 VS Profile 1	
	*Coef(SE)*	OR	*Coef(SE)*	OR	*Coef(SE)*	OR
Gender	-1.272(0.114) ^***^	0.032	-1.253(0.102) ^***^	0.286	-0.972(0.071)^***^	0.378
Age	0.171(0.034) ^***^	1.186	0.012(0.028)	1.012	0.028(0.020)	1.028
family structure	-0.262(0.103) ^*^	0.770	0.175(0.092)	1.191	-0.117(0.067)	0.890
The highest education level of the family	0.003(0.055)	1.003	-0.205(0.049) ^***^	0.815	-0.007(0.035)	0.993
Family occupational stratum	-0.095(0.063)	0.909	-0.165(0.058) ^**^	0.848	-0.021(0.037)	0.979

***p < .001, **p < .01, *p < .05

The gaming time could also predict the latent profile of the gaming disorder. Table 6 showed the results of multiple Logistic regression that with the healthy gamers as the reference group, with the increase of online gaming time, the OR of the impaired gamers, the impaired control-game priority gamers, and the gamers with disorder was 21%, 63.2%, and 61.1% higher, respectively. With the increase of stand-alone gaming time, the OR of these gamers in the above three profiles was 69.7%, 89.6%, and 70.4% higher, respectively. Additionally, with the increase of video gaming time, the OR of these gamers in the above three profiles was 82.9%, 3% and 74.5% higher, respectively.

**Table 6:** Multiple logistic regression results of online, stand-alone and video games in 4 latent profiles

Variable	Profile 2 VS Profile 1		Profile 3 VS Profile 1		Profile 4 VS Profile 1	
	*Coef(SE)*	OR	*Coef(SE)*	OR	*Coef(SE)*	OR
Online game	0.793(0.035) ^***^	2.210	0.490(0.034) ^***^	1.632	0.477(0.029) ^***^	1.611
Stand-alone game	0.992(0.045) ^***^	2.697	0.640(0.042) ^***^	1.896	0.533(0.039) ^***^	1.704
Video game	1.040(0.047) ^***^	2.829	0.708(0.043) ^***^	2.030	0.557(0.042) ^***^	1.745

***p < .001, **p < .01, *p < .05

## Discussion

The present study mainly focused on to investigating the GD profiles of adolescents with latent profiles analysis and to exploring the relationship between adolescents’ latent profiles and demographic variables –age, gender, family structure, the highest level of education of family members and family occupational stratum.

### LPA of GD

The LPA findings indicated that individuals can be classified into four-profile model, the present results align with Paschke et al. [29]. The profiles for healthy gamers (Profile 1), impaired control gamers (Profile 2), impaired control-game priority gamers (Profile 3), and gamers with disorder (Profile 4), which are four types of gamers with different severity and frequency thresholds. Different from previous studies that have generally focused only on the differences between the impaired and normal groups, we made additional distinctions between the different characteristics of individuals with GD. If inter-individual heterogeneity is ignored in a study, and people with GD are compared to normal controls as a homogeneous whole, it is possible that a risk of false-negative results, which may prevent the study from drawing meaningful conclusions and carrying out the appropriate clinical applications.

Our study showed that the 4-profile model had the best fit. Profile 1 group who showed to retain control while gaming without serious negative consequences. Profile 2 group showed impaired control, with rather increasing priority to gaming, and a lower level of functional impairment concerning their other symptoms. Profile 3 group, in addition to showing impaired control, also showed a significantly higher level of loss of increasing priority to gaming. Profile 4 group showed the highest levels of impaired control, increasing priority to gaming, and continued use despite harm) and functional impairment symptoms.

According to the highest score was found in the dimension of impaired control over gaming behavior, our study found impaired control as the core symptom of GD and its associated impairments, which is consistent with the findings of King et al [30]. When gamers are consistently preoccupied with thoughts about gaming, and when gamers have uncontrolled cravings for the game, these might be signs of impaired control over gaming [31, 32]. Neurobiologically, hypoactive prefrontal-striatal circuits are involved in cognitive control over the behaviors [33], which is an early stages of GD [34]. Studies on subjects with IGD proposed that Profile 1 could control their game pattern and keep their impulsivity control level [9]. On the contrary, IGD patients were often unable to correctly regulate gaming behaviors because of the failure of control systems [35, 36].

Profile 3 group showed higher intensity of presenting symptoms than profile 2 group on GD symptoms, especially gaming takes precedence over other life interests and increasing priority is given to gaming. In addition, the impaired control, increasing priority, and obvious functional impairment were higher than the threshold, but continuous gaming regardless of negative consequences was lower than the threshold. Profile 4 group showed higher probabilities of GD symptom than impaired control-game priority gamers on all symptoms, especially continuation or escalation of gaming despite the occurrence of negative consequences. The scores of gamers with disorder in all dimensions were high. These profiles are consistent with existing LPA data involving symptoms assessing GD.

### GD correlates

Multiple logistic regression analysis of this study showed that age, gender and weekly gaming time had significantly different effects on adolescents in different gaming profiles. We found that GD has been largely associated with being male, which is consistent with findings reported in previous studies, the GD prevalence was higher in male adolescents [37, 38]. The reason for this may be that men prefer competitive and confrontational activities in their choice of entertainment, and a high proportion of games of all kinds have these characteristics. Biologically, corticosteroid-limbic brain regions as well as others were activated to a greater extent by gaming cues in males compared to females [39]. Age was able to predict students belonging to the Profile 2, but it was not significant in predicting the Profile 3 and Profile 4. The reason could be that adolescents are in a stage of rapid physical and mental development, which is often characterized by uneven, insufficient, and fluctuating development. However, adolescents’ ability to control and manage their own impulses and behaviors also plays an important role in protecting themselves from addictions such as GD, and has a key impact on their overall physical and mental health. Being an only children may be more likely to develop into the Profile 2. The reason could be that only children like to seek peer interaction or find pleasurable experiences through online gaming. In addition, the level of education in the family may be related to the development of Profile 3, e.g., the level of education may modulate adolescents’ impaired control and increasing priority to gaming and thus influence the development of Profile 3. Consistent with previous findings [40], our study found that weekly game time was a risk factor for GD. The more time students spend on the gaming, the more disruption or displacement of normal routine and functioning, which eventually leads to GD. Therefore, gaming (include online, stand-alone and/or video games) for a long time may a significant predictor of GD in adolescents.

Although there are many studies [41, 42] on GD, this study conducted LPA in Chinese adolescents for the first time. The LPA four-profile model can help us further understand the population differences of GD, and the specific risk characteristics of each profile, thus taking more detailed and targeted measures. Health education may be given to Profile 1 and Profile 2. Short intervention may benefit Profile 3, especially the application of appropriate psychotherapy techniques for adolescents, such as timely feedback, motivation enhancement, self-efficacy enhancement, correction of misconception, promotion of behavior changes and provision of suggestions. For Profile 4, an enhanced brief intervention should be given, that is, after the initial brief intervention, a brief intervention will be carried out again, and they will be referred to the psychiatric department or addiction specialist for further diagnosis and treatment. In addition, the health triage service in China may help screen the problematic gaming behaviors of adolescents.

### Limitations

It is worth noting that this study has several limitations. First, we used a convenience sampling method to conduct this research in twelve schools in Xinjiang Uygur Autonomous Region, China, which should be validated in other regions of China in the future. Second, the instrument was self-reported and might have suffered from social desirability bias. Third, the current research did not compare the GDSQ-21 instrument with the gold standard (i.e., psychiatric clinical diagnostic interviews). therefore, the values of accuracy, specificity, and sensitivity could not be determined. Fourth, we used a cross-sectional approach, which may be unable to infer causal relationships between variables. Therefore, further research is needed to explore longitudinal relationships.

## Conclusions

To the best of our knowledge, this study is the first to investigate the symptom pattern of GD in Chinese adolescents. This study used a person-centered approach (LPA) to derive four distinct groups of gamers based on GDSQ-21 symptoms and the relationship between latent classes and background variables i.e. age, gender, family structure, and weekly gaming time of the subjects. In addition, the current findings provide a reference point for timely and effective clinical screening of individuals and the implementation of different intervention and prevention strategies. This may help our medical practitioners and other professionals closely involved with adolescents (e.g., teachers) to identify individuals with high levels of symptoms of GD and to triage them for services.

### Electronic supplementary material

Below is the link to the electronic supplementary material.

**Additional file 1**: Supplementary Table S1. The conditional mean value of each item after LPA.

## Data Availability

The datasets used and/or analyzed during the present study are available from the corresponding author on reasonable request.

## Abbreviations

IGD: internet gaming disorder
DSM-5: Diagnostic and Statistical Manual of Mental Disorders
ICD-11: International Classification of Diseases 11th Revision
GD: gaming disorder
GDSQ-21: Gaming Disorder Symptom Questionnaire-21
LPA: Latent profile analysis
AIC: Akaike information criteria
BLRT: Bootstrap; Likelihood Ratio Test
OR: odds ratio
