# Supplementary material for: The latent profile analysis of Chinese adolescents’ gaming disorder: examination and validation
Source: BMC Psychiatry. 2023 Nov 13;23:833. doi: 10.1186/s12888-023-05320-8 (PMC10644538; doi:10.1186/s12888-023-05320-8)
Supplement: Supplementary file 1 — Additional file 1: Supplementary Table S1. The conditional mean value of each item after LPA. [file 12888_2023_5320_MOESM1_ESM.docx]

**Supplementary Table S1** Conditional mean value of each item after LPA

| **Item** | **Conditional mean** | | | |
| --- | --- | --- | --- | --- |
|  | **Profile 1** | **Profile 2** | **Profile 3** | **Profile 4** |
| Item 1 | 1.172 | 2.460 | 2.583 | 3.173 |
| Item 2 | 0.629 | 2.150 | 2.541 | 3.160 |
| Item 3 | 0.526 | 2.147 | 2.557 | 3.171 |
| Item 4 | 0.387 | 1.931 | 2.575 | 3.186 |
| Item 5 | 0.245 | 1.424 | 2.387 | 2.871 |
| Item 6 | 0.257 | 1.342 | 2.399 | 2.893 |
| Item 7 | 0.266 | 1.241 | 2.501 | 3.060 |
| Item 8 | 0.197 | 0.815 | 2.367 | 2.788 |
| Item 9 | 0.081 | 0.389 | 2.137 | 2.546 |
| Item 10 | 0.286 | 1.123 | 2.497 | 2.956 |
| Item 11 | 0.089 | 0.397 | 2.292 | 2.622 |
| Item 12 | 0.098 | 0.427 | 2.179 | 2.691 |
| Item 13 | 0.104 | 0.404 | 2.192 | 2.610 |
| Item 14ab | 0.028 | 0.255 | 0.699 | 2.889 |
| Item 15ab | 0.031 | 0.220 | 0.509 | 2.959 |
| Item 16ab | 0.032 | 0.232 | 0.472 | 2.984 |
| Item 17ab | 0.032 | 0.212 | 0.434 | 2.556 |
| Item 18ab | 0.006 | 0.087 | 0.262 | 2.389 |
| Item 19ab | 0.028 | 0.218 | 0.322 | 2.426 |
| Item 20ab | 0.034 | 0.213 | 0.373 | 2.276 |
| Item 21ab | 0.024 | 0.158 | 0.293 | 2.462 |
